# Exercise training reduces resting heart rate via downregulation of the funny channel HCN4

**DOI:** 10.1038/ncomms4775

**Published:** 2014-05-13

**Authors:** Alicia D’Souza, Annalisa Bucchi, Anne Berit Johnsen, Sunil Jit R.J. Logantha, Oliver Monfredi, Joseph Yanni, Sukhpal Prehar, George Hart, Elizabeth Cartwright, Ulrik Wisloff, Halina Dobryznski, Dario DiFrancesco, Gwilym M. Morris, Mark R. Boyett

**Affiliations:** 1 Institute of Cardiovascular Sciences, University of Manchester, Manchester M13 9NT, UK; 2 Department of Biosciences, University of Milano, Milano 20133, Italy; 3 Department of Circulation and Medical Imaging, Norwegian University of Science and Technology, Trondheim 7491, Norway; 4 These authors contributed equally to this work

## Abstract

Endurance athletes exhibit sinus bradycardia, that is a slow resting heart rate, associated with a higher incidence of sinus node (pacemaker) disease and electronic pacemaker implantation. Here we show that training-induced bradycardia is not a consequence of changes in the activity of the autonomic nervous system but is caused by intrinsic electrophysiological changes in the sinus node. We demonstrate that training-induced bradycardia persists after blockade of the autonomous nervous system *in vivo* in mice and *in vitro* in the denervated sinus node. We also show that a widespread remodelling of pacemaker ion channels, notably a downregulation of HCN4 and the corresponding ionic current, *I*_f_. Block of *I*_f_ abolishes the difference in heart rate between trained and sedentary animals *in vivo* and *in vitro*. We further observe training-induced downregulation of Tbx3 and upregulation of NRSF and miR-1 (transcriptional regulators) that explains the downregulation of HCN4. Our findings provide a molecular explanation for the potentially pathological heart rate adaptation to exercise training.

## Abstract (web summary)

Endurance athletes are known to have a low resting heart rate. Here, D'Souza *et al.* propose that training-induced bradycardia is the result of electrophysiological changes in the sinus node, challenging the classical view that training-induced bradycardia is caused by increased activity of the autonomic nervous system.

## Graphical abstract

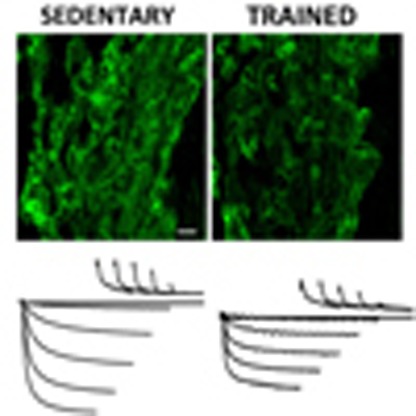

Athletes are considered the healthiest members of society and yet, paradoxically, the incidence of arrhythmias, ranging from the benign to the pathological, is known to be higher in athletes, especially in elderly athletes with a lifelong training history^1^,^2^,^3^,^4^,^5^,^6^,^7^. Emerging data suggest that long-term training for and competing in extreme endurance events such as marathons, ultra-marathons, ironman distance triathlons and long distance bicycle races may induce pathological remodelling of the heart and potentially create a substrate for arrhythmogenesis^8^. Furthermore, there is a rise in the number of individuals participating in gruelling ultra-endurance events. For example, each year there are >500 marathon races in Europe and America, and >1 million participants and this figure is projected to rise by at least 5% each year^9^. Sinus bradycardia, defined by a resting heart rate <60 beats min^−1^, is the most frequent rhythm disturbance in response to exercise training; the heart rate can be ~30 beats min^−1^ and even lower at night^5^,^10^,^11^. Although the bradycardia is usually a benign physiological adaptation (to maintain a normal cardiac output and blood pressure despite the training-induced increase in stroke volume), it can become a pathological condition resembling sinus node disease^5^,^6^,^7^. The most compelling evidence of a link between training and sick sinus syndrome comes from a study of former professional cyclists^7^. Their average heart rate was lower, a maximal RR interval of >2.5 s was more common, sick sinus syndrome was more frequent and pacemaker implantation for bradyarrythmias was more frequent relative to a control group with matched cardiac risk factors^7^. Similarly, a high incidence of pacemaker implantation has been reported in elderly marathon runners^12^. The training-induced bradycardia is widely attributed to the autonomic nervous system–an increase in vagal tone induced by training^13^,^14^. However, slower resting heart rates are observed in athletes even after complete block of the autonomic nervous system^15^. We hypothesized that, instead, the training-induced bradycardia is primarily because of an intrinsic change in the sinus node, the pacemaker of the heart, in particular, to a remodelling of the ion channels that govern pacemaking. We show that this is the case. An improved understanding of the athletes’s heart could ultimately lead to better-informed lifestyle choices for the athlete.

## Results

### Exercise training produces an intrinsic sinus bradycardia

**Figure 1:**
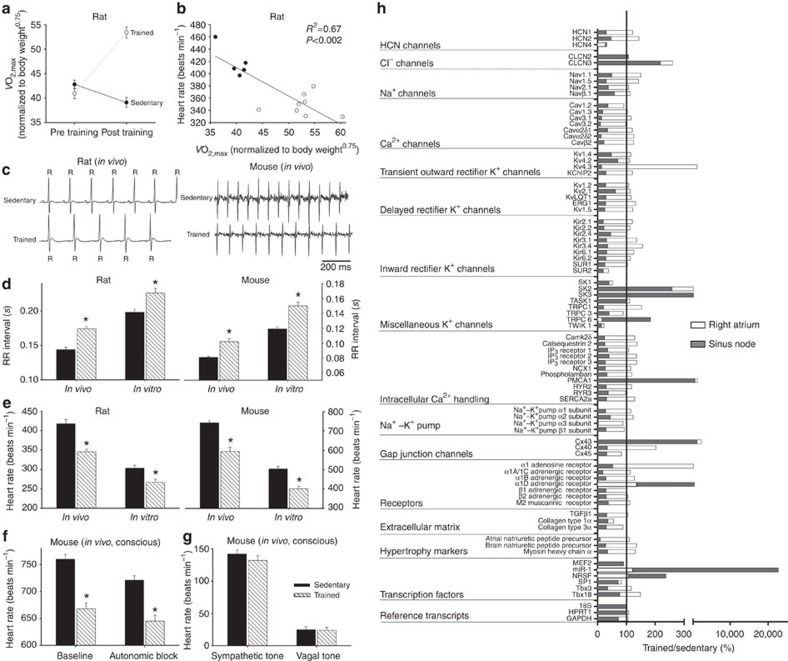
Exercise training induces sinus bradycardia and remodelling of the sinus node. (**a**) Increase in 
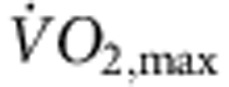
 in the rat following the 12-week training period. Normalized 
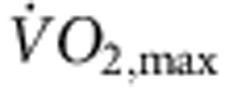
 of sedentary and trained rats before and after the 12-week training period shown (*n*=11 per group). In all bar graphs: black bars represent data from sedentary animals and hatched bars represent data from trained animals. (**b**) Significant correlation between heart rate *in vitro* and normalized 
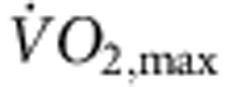
 in sedentary and trained rats (measured after 12-week training period). Each point corresponds to a different animal. Data fit with a straight line by linear regression (*n*=5/9; *R*^2^ and *P* values shown). (**c**) Representative ECG traces obtained from sedentary and trained (unrestrained and conscious) rats and mice demonstrating longer RR intervals in trained animals. (**d**,**e**) Mean (+s.e.m.) RR intervals (**d**) and corresponding heart rates (**e**) measured *in vivo* (rats, *n*=5/9; mice, *n*=6/8) and *in vitro* (from isolated sinus node preparations; rats, *n*=5/9; mice, *n*=6/7) in sedentary and trained animals. (**f**) Mean (+s.e.m.) heart rate measured *in vivo* in conscious mice at baseline (*n*=9/11) and after complete autonomic block with propranolol and atropine (*n*=7/14). (**g**) Mean (+s.e.m.) sympathetic tone and vagal tone in conscious sedentary and trained mice (*n*=6/6). (**h**) Expression of transcripts in the sinus node (grey bars) and atrial muscle (open bars) of trained rats as a percentage of that of sedentary rats. The vertical line corresponds to 100%, that is, no change. Values <100% correspond to a decrease on training and >100% an increase. Student’s *t*-test used to test differences between data from trained and sedentary animals. Normal distribution of data was tested using the Shapiro–Wilk *W*-test and equal variance was tested using the *F*-test. When the null hypothesis of normality and/or equal variance was rejected, the non-parametric Mann–Whitney *U*-test was used. *P<0.05.

Rats were trained for 12 weeks (1 h per day, 5 days per week) by aerobic interval training (uphill running), alternating between 4 min at 85–90% of the maximum O_2_ uptake (
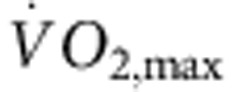
) and 2 min active recovery at 50% of 
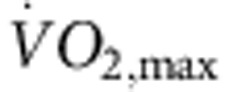
. As expected, in the trained, but not sedentary, rats there was an increase in 
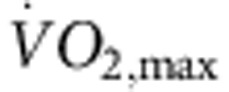
 (Fig. 1a). The electrocardiogram (ECG) was recorded *in vivo* (from unrestrained and conscious rats; Fig. 1c) and, as with human athletes, there was a resting bradycardia in the trained rats: the RR interval (interval between heart beats; Fig. 1c,d) of the rats at rest was significantly longer than that of age- and sex-matched sedentary rats; the corresponding heart rate (Fig. 1e) was significantly slower than that of the sedentary rats. The training-induced bradycardia could be the result of altered autonomic nerve activity to the heart (for example, increased vagal tone) or a change in the intrinsic properties of the sinus node. To distinguish between the two hypotheses, we measured the cycle length (equivalent to the RR interval) in the denervated sinus node (isolated *in vitro* preparation used) and this too was significantly longer in the trained rats (Fig. 1d); the corresponding rate was significantly slower (Fig. 1e). In the rat, there was a significant correlation between the rate *in vitro* and 
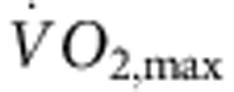
 (Fig. 1b).

Experiments were also carried out in the mouse—mice were trained for 4 weeks (1 h twice a day, 7 days per week) by swimming, and in the trained mice there was once again a resting bradycardia *in vivo* in the conscious mouse (Fig. 1c–e). In the mouse, the two opposing hypotheses above were tested *in vitro* and also *in vivo*. *In vitro* (in the denervated sinus node), the cycle length was significantly longer and the rate was significantly slower in the trained mice, as in the case of the rat (Fig. 1d,e). *In vivo* (conscious mouse), the heart rate (from this point onwards, only heart rate will be reported) was still significantly slower in the trained mice after complete autonomic block by propranolol and atropine (Fig. 1f). During the course of these experiments, it was possible to measure sympathetic and vagal tone—sympathetic tone was taken as the change (decrease) in heart rate on blocking sympathetic nerve activity with propranolol (in the presence of atropine) and vagal tone was taken as the change (increase) in heart rate on blocking vagal nerve activity with atropine (in the presence of propranolol). Sympathetic and vagal tone were the same in the trained and sedentary mice (Fig. 1g); there was no evidence of an increase in vagal tone following training. All of these findings in the rat and mouse demonstrate that the training-induced resting bradycardia is predominantly the result of a change in the intrinsic properties of the sinus node.

### Training-induced ion channel remodelling in the sinus node

The most likely change is of the ionic mechanisms underlying the pacemaker activity of the sinus node, and a targeted transcriptomics approach was used to investigate this. Tissue samples (1 mm) were taken from the sinus node (and neighbouring right atrial muscle) of sedentary and trained rats. The expression of mRNAs for 83 ion channels and related molecules was measured by quantitative PCR (qPCR). Expression of all transcripts was expressed relative to a housekeeper (reference transcript). Three widely used housekeepers (*18S, HPRT* and *GAPDH*) were evaluated using the algorithm geNorm within RealTime StatMiner (Integromics, USA) and *18S* was considered the best. Although the mRNA expression data shown are relative to 18S, it was confirmed that qualitatively similar data were obtained using the other housekeepers. In Fig. 1h and Supplementary Table 1, expression in the trained rats is shown as a percentage of that in the sedentary rats. In the sinus node of the trained rats, of the transcripts targeted, a few were upregulated or unchanged and the majority were significantly downregulated (Fig. 1h; Supplementary Tables 1 and 2). There was a widespread downregulation of mRNAs for hyperpolarization-activated cyclic nucleotide-gated (HCN), Na^+^, Ca^2+^ and K^+^ channels, intracellular Ca^2+^-handling molecules, components of the Na^+^–K^+^ pump, gap junctions, autonomic receptors and components of the extracellular matrix (Fig. 1h; Supplementary Table 1). In contrast, few of the measured mRNAs were significantly altered in the atrial muscle (Fig. 1h).

**Figure 2:**
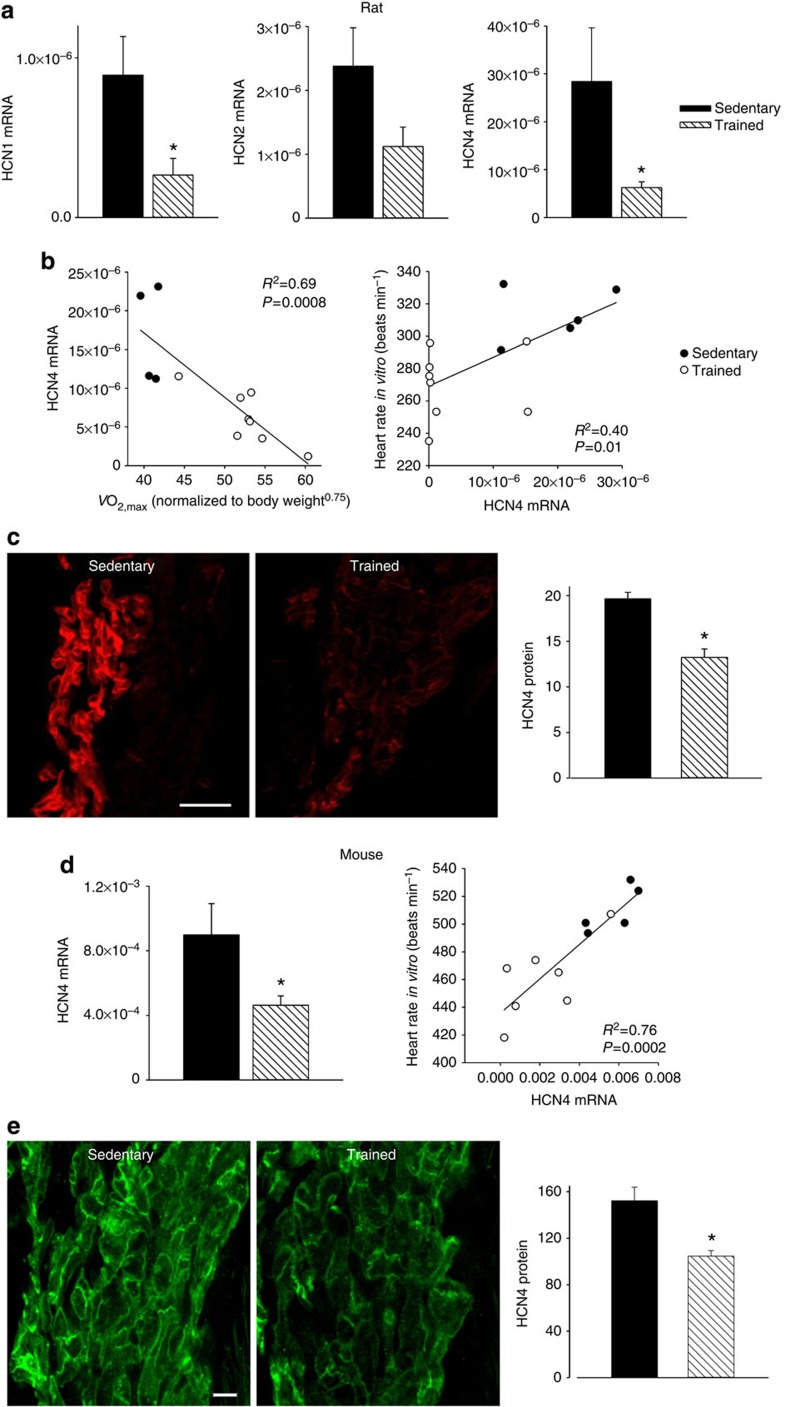
HCN channels in sinus node are downregulated by training. (**a**) Downregulation of mRNA for HCN channels in trained rats. Mean (+s.e.m.) expression of mRNA for HCN1, HCN2 and HCN4 (normalized to expression of 18S) in the sinus node of sedentary and trained rats is shown (*n*=5/8; FDR-corrected Limma test). (**b**) Significant correlations between HCN4 mRNA and normalized 
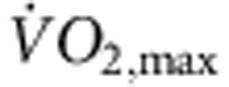
 (left) and heart rate *in vitro* and HCN4 mRNA (right) in sedentary and trained rats. Each point corresponds to a different animal. In all scatter plots: filled circles represent data from sedentary animals, and open circles represent data from trained animals. Data fit with straight lines by linear regression (*n*=4/8; *R*^2^ and *P* values shown). (**c**) Downregulation of HCN4 protein in trained rats. Representative images of HCN4 immunolabelling (red signal) in the sinus node of sedentary and trained rats are shown in the left and middle panels, respectively (scale bar, 100 μm). The right panel shows the mean (+s.e.m.) HCN4 protein expression quantified using ImageJ from the NIH (*n*=4). (**d**) Downregulation of mRNA for HCN4 in trained mice. The left panel shows the mean (+s.e.m.) expression of HCN4 mRNA (normalized to expression of 18S) in the sinus node of sedentary and trained mice (*n*=6/7) and the right panel shows a significant correlation between heart rate *in vitro* and HCN4 mRNA in sedentary and trained mice. Each point corresponds to a different animal. Data fit with a straight line by linear regression (*n*=5/7; *R*^2^ and *P* values shown). (**e**) Downregulation of HCN4 protein in trained mice. Representative images of HCN4 immunolabelling (green signal) in the sinus node of sedentary and trained mice are shown in the left and middle panels, respectively (scale bar, 10 μm). The right panel shows mean (+s.e.m.) HCN4 protein expression (*n*=4). Student’s *t*-test used to test differences between data from sedentary and trained animals. Normal distribution of data was tested using the Shapiro–Wilk *W*-test and equal variance was tested using the *F*-test. When the null hypothesis of normality and/or equal variance was rejected, the non-parametric Mann–Whitney *U*-test was used. *P<0.05.

There are two principal pacemaker mechanisms in the sinus node: the Ca^2+^ clock and the membrane clock^16^. In the trained rat, there was a significant downregulation of mRNA for various components of the Ca^2+^ clock such as RyR2 (Supplementary Fig. 1). There was also a trend for a decrease in RyR2 expression at the protein level, but the decrease did not reach significance (Student’s *t-*test, *P*=0.06; Supplementary Fig. 1c,d). In the isolated sinus node, functioning of the Ca^2+^ clock can be assessed by measuring the decrease in rate on incapacitating the Ca^2+^ clock with 2 μM ryanodine. In the trained rat, there was also a trend for a decrease in Ca^2+^ clock function, but again the decrease did not reach significance (Student’s *t-*test*, P*=0.056; Supplementary Fig. 1e). In contrast, in the trained mouse, there were no indications of a downregulation of the Ca^2+^ clock (Supplementary Fig. 1). In summary, the data do not unequivocally show a role for the Ca^2+^ clock (although a role in the trained rat cannot be excluded). The most important component of the membrane clock is the funny current (*I*_f_)^17^, for which HCN1 and in particular HCN4 are responsible. Figure 2a shows in more detail the downregulation of HCN mRNAs in the sinus node of the trained rats. In the rat, there were significant correlations between HCN4 mRNA expression and 
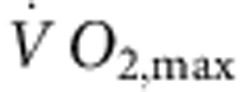
 (although there were significant correlations between other transcripts and 
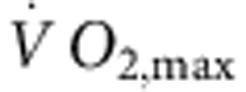
, *R*^2^ was highest for HCN4; Supplementary Table 3), as well as heart rate *in vitro* and HCN4 mRNA expression (Fig. 2b). This suggests that fitness (as measured by 
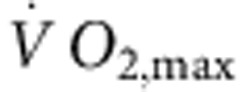
), HCN4 mRNA and pacemaking are linked. Protein levels of HCN4 were assessed using immunohistochemistry (western blot unsuitable)—there was a significant downregulation of HCN4 protein (Fig. 2c) consistent with the mRNA data. In the mouse, there was also a downregulation of HCN4 mRNA and protein in the sinus node following training and also a significant correlation between heart rate and HCN4 mRNA expression (Fig. 2d,e). A correlation between downregulation of HCN4 protein and rate slowing agrees with previous data from cardiac-specific HCN4 knockout mice^18^. The coefficient of determination (*R*^2^) for the correlation between heart rate and HCN4 mRNA expression was 0.4 (rat) and 0.76 (mouse); this suggests that at least 40% and 76%, respectively, of the variation in heart rate can be attributed to the change in HCN4 mRNA (if there are inaccuracies in the data, the percentages could be higher).

**Figure 3:**
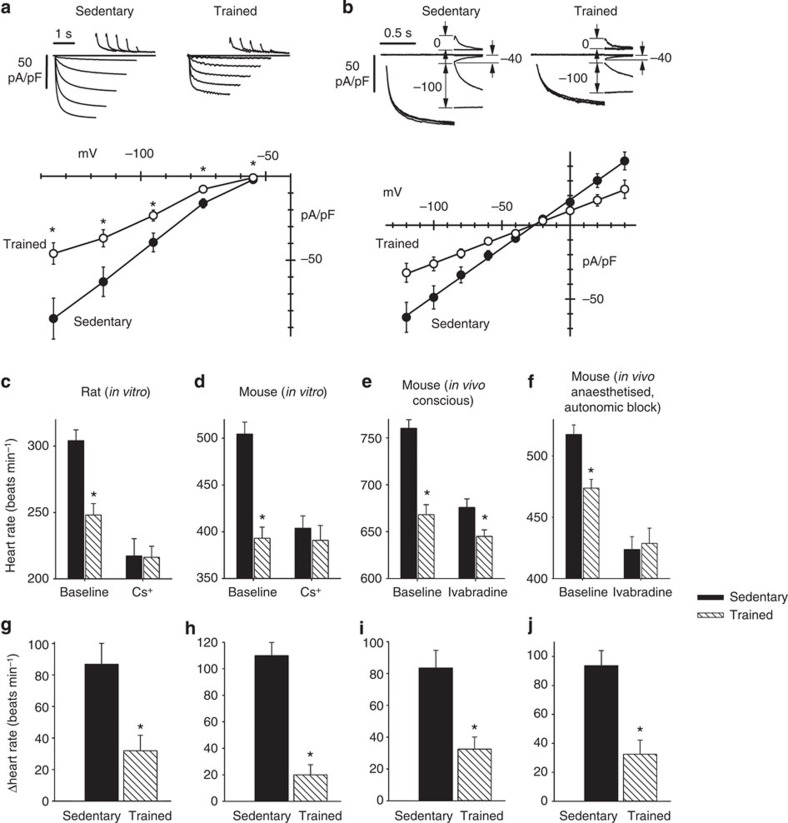
Downregulation of *I*_f_ in the sinus node can explain the training-induced resting bradycardia. (**a**) Density of *I*_f_ is reduced in trained mice. Top, representative *I*_f_ traces, normalized to cell capacitance, during steps to -55 to -135 mV (20 mV increments; holding potential −35 mV) from single sinus node cells isolated from sedentary (left) and trained (right) mice. Bottom, mean (±s.e.m.) *I*_f_ IV curves recorded at steady state from sedentary (filled circles; *n*=17/5 cells per mice) and trained (open circles; *n*=18/5) mice. Student’s *t*-test used to test differences between sedentary and trained mice; *P<0.05. (**b**) Whole-cell conductance of *I*_f_ is reduced in trained mice. Top, sample current traces recorded with the protocol used to measure the fully activated IV relationship of *I*_f_ from single sinus node cells isolated from sedentary (left) and trained (right) mice. Conditioning activating/deactivating voltages were −125 and −35 mV, and pairs of steps to the same test voltage were applied in sequence from these two levels; records shown for test voltages of −100, −40 and 0 mV. Bottom, mean (±s.e.m.) fully activated I/V relationship, normalized to cell capacitance, from sedentary (filled circles; *n*=9/5 cells per mice) and trained (open circles; *n*=11/5) mice. Linear fit of data (straight lines) yielded conductances of 653 and 356 pS/pF and reversal potentials of -26.3 and -27.3 mV for sedentary and trained mice. The two curves are statistically different (*F*-test; *P*<0.05). (**c**,**d**), Spontaneous rate of isolated rat (**c**; *n*=7) and mouse (**d**; *n*=6/7) sinus node preparations from sedentary and trained animals before and after *I*_f_ block with Cs^+^. (**e**,**f**) Heart rate of sedentary and trained mice when conscious (**e**; *n*=9/13) and when anaesthetized and after autonomic block (**f**) before and after *I*_f_ block with ivabradine (*n*=7/14). (**g**–**j**) Decrease in heart rate (beats min^−1^) on blocking *I*_*f*_ in corresponding experiment above (**c**–**f**). In c-j, means+s.e.m. shown. Student’s *t*-test used to test differences between sedentary and trained animals. Normal distribution of data tested using Shapiro–Wilk *W*-test and equal variance tested using *F*-test. When null hypothesis of normality and/or equal variance was rejected, non-parametric Mann–Whitney *U*-test used. *P<0.05.

To test directly whether *I*_f_ is affected by training, sinus node cells were isolated from sedentary and trained animals by enzymatic dispersion and were analysed by patch-clamp in the whole-cell configuration. Experiments were carried out on the mouse, a popular species for patch clamp of sinus node cells. We first compared *I*_f_ densities recorded in sinus node cells from sedentary (*n*=17 cells from 5 mice) and trained (*n*=18/5) mice. Currents recorded in the range −55 to −135 mV were normalized to cell capacitance to obtain current density. The mean cell capacitance was 27.1±1.4 pF (*n*=17/5) and 28.8±1.1 pF (*n*=18/5) in sedentary and trained mice, respectively (not significantly different, Student’s *t*-test, *P*>0.05). In Fig. 3a, sample current traces and mean current–voltage (IV) curves are shown. The *I*_f_ density was reduced in trained mice by ~47% over the whole potential range. We next investigated whether the *I*_f_ density reduction in trained mice involves changes in the current kinetics or in the whole-cell conductance, or both. In Supplementary Fig. 2, sample traces from sedentary (left) and trained (right) mice used to measure current kinetics are shown. Analysis of the activation curve (Supplementary Fig. 2b) and of activation/deactivation time constant curves (Supplementary Fig. 2c) shows that the kinetic properties of *I*_f_ were not modified by training. On the other hand, as shown in Fig. 3b, analysis of the fully activated IV curve clearly shows a reduction of the whole-cell *I*_f_ conductance in trained mice. Linear fitting of the fully activated IV relations, normalized to cell capacitance, yielded *I*_f_ conductances of 653 and 356 pS/pF for sedentary and trained animals, (*F*-test, *P*<0.05). These data suggest that training reduces substantially (~45%) the expression of *I*_f_ channels on the membrane of sinus node cells from trained animals.

### Role of training-induced downregulation of HCN channels

The role of *I*_f_ was assessed *in vitro* and *in vivo* by blocking *I*_f_. *In vitro, I*_f_ was blocked by 2 mM Cs^+^ in the isolated sinus node from both rat and mouse^19^. *In vivo*, *I*_f_ was blocked by administration of ivabradine (S16257-2; 6 mg kg^−1^ per body weight^20^), which dose-dependently reduces heart rate in humans^21^ and animals^22^,^23^; these experiments were performed in mice only, but they were performed in conscious mice and in anaesthetized mice after complete autonomic block. Absolute heart rates before and after block of *I*_f_ are shown in Fig. 3c–f, and increments in heart rate on block of *I*_f_ are shown in Fig. 3g–j. In every case, block of *I*_f_ by Cs^+^ or ivabradine produced a decrease in heart rate as expected (Fig. 3c–f), but the decrease in heart rate was significantly less in trained animals (Fig. 3g–j). This is consistent with the downregulation in HCN channels and *I*_f_ in the sinus node in the trained animals. *In vitro*, block of *I*_f_ by Cs^+^ abolished the heart rate difference between trained and sedentary rats and mice (Fig. 3c,d). *In vivo*, block of *I*_f_ by ivabradine greatly reduced the difference in the conscious mouse and abolished it in the anaesthetized mouse after autonomic block (Fig. 3e,f). In the case of the mouse, the data can be analysed in another manner. In the mouse, training reduced *I*_f_ by ~45%. Pharmacological block of *I*_f_ by a similar amount should produce a similar bradycardia as training. In isolated mouse sinus node cells, 2 mM Cs^+^ blocked *I*_f_ by 79%, 70% and 56% at −90, −70 and −50 mV, respectively (Supplementary Fig. 3). The data show voltage-dependent block in agreement with our previous results from rabbit sinus node cells^24^. The degree of block of *I*_f_ by Cs^+^ at diastolic potentials (around −50 mV) is comparable to that caused by training; and Fig. 3d shows that 2 mM Cs^+^ caused a similar bradycardia as training as expected. These results point to downregulation of *I*_f_ as the predominant mechanism underlying exercise training-induced bradycardia.

### Mechanisms underlying the HCN4 downregulation in exercise

**Figure 4:**
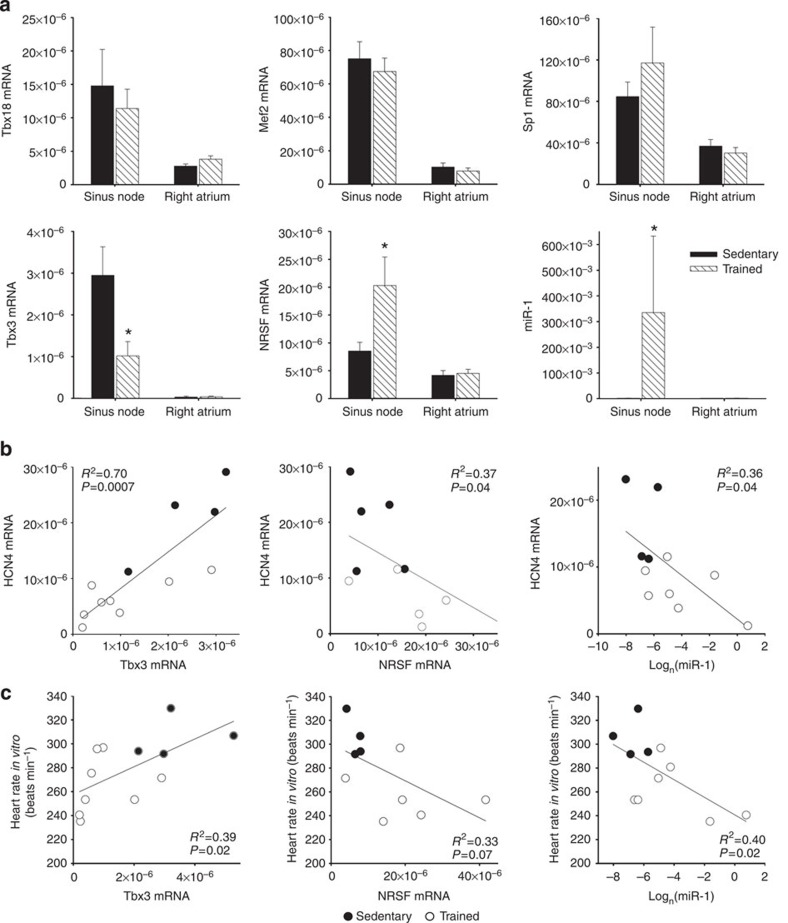
Three regulators of HCN4 in the sinus node are altered by training. (**a**) No changes in the expression of three regulators of HCN4 in the sinus node in trained rats, but significant changes in another three. Mean (+s.e.m.) mRNA expression of Tbx18 (sinus node, *n*=6/6; right atrial muscle, *n*=7/6), Mef2 (sinus node, *n*=6/5; right atrial muscle, *n*=7/6), Sp1 (sinus node and right atrial muscle, *n*=6/6), Tbx3 (sinus node, *n*=5/8; right atrial muscle, 8/7), NRSF (sinus node and right atrial muscle, *n*=7/6) and miR-1 (sinus node, *n*=4/7; right atrial muscle, *n*=6/5) (normalized to expression of 18S in the case of mRNAs and RNU1A1 in the case of miR-1) in the sinus node and right atrial muscle of sedentary and trained rats shown. (**b**,**c**) Significant correlations between HCN4 mRNA (**b**) and heart rate *in vitro* (**c**) and Tbx3 mRNA, NRSF mRNA and miR-1 in sedentary and trained rats. Each point corresponds to a different animal. Data fit with straight lines by linear regression (*n*=4–8, *R*^2^ and *P* values shown). Student’s *t*-test used to test differences between data from sedentary and trained rats. Normal distribution of data was tested using the Shapiro–Wilk *W*-test and equal variance was tested using the *F*-test. When the null hypothesis of normality and/or equal variance was rejected, the non-parametric Mann–Whitney *U*-test was used. *P<0.05.

**Figure 5:**
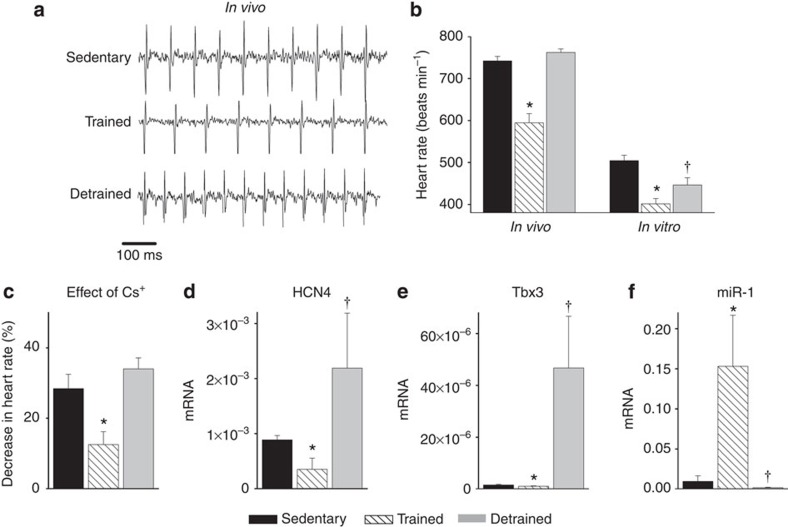
Sinus bradycardia and sinus node remodelling in the mouse are reversed by detraining. (**a**) Representative ECG traces obtained from sedentary, trained and detrained mice (unrestrained and conscious). (**b**) Mean (+s.e.m.) heart rate measured *in vivo* (*n*=7/8/5) and *in vitro* (from isolated sinus node preparations; *n*=7/8/5) in sedentary, trained and detrained mice. (**c**) Restoration of the contribution of *I*_f_ to pacemaking in the detrained mouse. Mean (+s.e.m.) percentage decrease in heart rate of isolated sinus node preparations from sedentary, trained and detrained mice on blocking *I*_f_ using 2 mM CsCl shown (*n*=6/7/4). (**d**–**f**) Restoration of normal levels of HCN4 and Tbx3 mRNA and miR-1 in the sinus node in the detrained mouse. Mean (+s.e.m.) expression of HCN4 and Tbx3 mRNA (normalized to 18S) and miR-1 (normalized to RNU1A1) in the sinus node of sedentary, trained and detrained mice shown (*n*=4). Student’s *t*-test used to test differences. Normal distribution of data was tested using the Shapiro–Wilk *W*-test and equal variance was tested using the *F*-test. When the null hypothesis of normality and/or equal variance was rejected, the non-parametric Mann–Whitney *U*-test was used. *P<0.05, trained versus sedentary mice; †*P*<0.05, detrained versus trained mice.

What regulates the response of the sinus node, in particular of HCN4, to training? The transcription factors, Tbx3, Tbx18, Mef2c, NRSF and Sp1, as well as microRNA-1 (miR-1), have been reported to regulate HCN4 mRNA levels^25^,^26^,^27^,^28^,^29^,^30^,^31^. For example, the transcription factors, Tbx3 and Tbx18, are known to control the pacemaking phenotype of the sinus node, and ectopic expression of Tbx3 and Tbx18 in working myocardium causes an upregulation of HCN4 and a switch to a sinus node phenotype^25^,^28^,^32^. In the rat, following training, there was no significant change in Tbx18, Mef2c and Sp1 mRNAs in the sinus node, but there was a significant downregulation of Tbx3 mRNA and upregulation of NRSF mRNA and miR-1 (Fig. 4a). The changes in Tbx3, NRSF and miR-1 are appropriate to explain the downregulation of HCN4 in the sinus node following training^25^,^26^,^27^,^30^,^31^. In the rat, it was investigated whether expression of mRNAs for HCN4, Ca^2+^-handling proteins and voltage-gated Ca^2+^ channels is correlated with the expression of Tbx3 mRNA, NRSF mRNA and miR-1. HCN4 was significantly correlated with Tbx3, NRSF and miR-1; *R*^2^ was highest for Tbx3 (Fig. 4b and Supplementary Table 4). None of the other targets tested were significantly correlated with NRSF and miR-1, but other targets, including *RyR2*, were significantly correlated with *Tbx3*, although *R*^2^ was less than for HCN4 (Supplementary Table 4). In the rat, there were significant correlations between heart rate and expression of Tbx3 mRNA, NRSF mRNA and miR-1 (Fig. 4c). The downregulation of Tbx3 mRNA and upregulation of miR-1 were observed in the mouse as well as the rat (Fig. 5e,f). Although the significant correlations of HCN4 with Tbx3, NRSF and miR-1 are suggestive, they do not prove a causative link for which further work is required (for example, use of genetically modified mice).

### Sinus node remodelling reverses on exercise cessation

The effects of detraining were studied in the mouse: mice were trained as described above and then the mice were detrained (they were sedentary) for 2 weeks. Figure 5a,b shows that the resting bradycardia was reversed—the heart rate *in vivo* returned to the pre-training level and the spontaneous rate *in vitro* was partially restored. Detraining also restored the response to block of *I*_f_, HCN4 and Tbx3 mRNA expression, and miR-1 expression, although intriguingly there appeared to be a rebound beyond the pre-training level in all cases (Fig. 5c–f).

## Discussion

This study is the first to show that the heart rate adaption to exercise training is not the result of changes in autonomic tone as previously thought^13^,^14^, and instead is primarily the result of a training-induced remodelling of the sinus node; of particular importance is a downregulation of HCN4 mRNA and protein, perhaps driven by Tbx3, NRSF and miR-1, and a consequent decrease in the density of *I*_f_.

There are reports of severe bradycardia in elite human athletes—on the internet one can find reports (non-peer-reviewed) that the resting heart rate of elite cyclists when race fit can be ~30 beats min^−1^. Jensen-Urstad *et al.*^10^ reported night time heart rates of <30 beats min^−1^ in male elite runners. A heart rate of ~30 beats min^−1^ is <50% of the heart rate of sedentary individuals (~70 beats min^−1^). In the present study, the heart rate of the trained rats and mice was ~26% and ~20%, respectively, lower than the heart rate of sedentary animals. This decrease is less than in elite human athletes. However, severe bradycardia in human athletes is uncommon. The heart rate of exercise-trained human subjects in various studies reviewed by Boyett *et al.*^15^ varied between 52 and 58 beats min^−1^, ~17–26% lower than the heart rate of sedentary individuals, a reduction similar to that observed in the animal models in the present study.

Some of the major conclusions of this study are based on qPCR analysis of mRNA expression in tissue biopsies (Fig. 1h). The sinus node is a heterogeneous tissue and, as well as nodal cells, contains fibroblasts, vascular smooth muscle cells and neuronal tissue. If the non-myocyte content of the biopsies used in this study was substantial, this will have consequences for the interpretation of the expression data summarized in Fig. 1h. In the human, it is known that up to 80% of myocardial cell volume (or ~90% of cell mass) is made up of cardiomyocytes with non-myocyte cells accounting for 10% (90–95% of which are fibroblasts)^33^. Therefore, it is likely that the predominant population of cells in the biopsies, by volume and mass at least, are cardiomyocytes. Fibroblasts are expected to be the largest source of non-myocyte mRNA. Fibroblasts are inexcitable and largely lack inward current carrying ion channels, such as HCN, Na^+^ and Ca^2+^ channels^34^,^35^. However, fibroblasts do express some K^+^ channels^34^. The content of neuronal mRNA in the biopsies should be minimal, because mRNA will be located in the rough endoplasmic reticulum next to the nucleus and the neuronal cell bodies are located either next to the spinal cord (sympathetic ganglia) or in the fat pads on the back of the atria (parasympathetic ganglia), neither of which would be present in the biopsies. In our study of the rat atrioventricular node^36^, we reported that Na_v_1.3 is expressed in neurones. Of course, Na_v_1.5 is expressed in cardiomyocytes. In similar biopsies of rat sinus node and atrial muscle to that used in the present study, we have previously reported that Na_v_1.5 mRNA is ~477x and ~1150x, respectively, which is more abundant than Na_v_1.3 mRNA^37^. This confirms that the content of neuronal mRNA in the biopsies is negligible. We compared the relative abundance of specific markers of different cell types using qPCR in sinus node biopsies from five 12-week-old C57BL6J mice. SERCA2a (Qiagen, QT0014912) was used as a specific marker for cardiomyocytes; vimentin (Qiagen, QT00159670) was used as a marker for fibroblasts; calponin 1 (Cnn1) was used as a marker of vascular smooth muscle cells^38^,^39^; and ubiquitin carboxy-terminal hydrolase L1 (Uchl1) was used as a neuronal marker^38^,^39^. SERCA2a expression was ~6-fold higher than that of vimentin, ~18-fold higher than that of Cnn1 and ~189-fold higher than that of Uchl1 (Supplementary Fig. 4). In summary, the data suggest that the expression data in Fig. 1h will largely reflect expression in cardiomyocytes, but the possibility that some data reflect changes in cells other than cardiomyocytes cannot be excluded.

Although the present study emphasizes the importance of the training-induced downregulation of HCN4 and *I*_f_, there is a widespread remodelling of the sinus node (Fig. 1h) and it is not possible to exclude the involvement of other pacemaker mechanisms. It is possible that other aspects of the remodelling of the sinus node following training will also impact on pacemaking and three examples will be given. In the trained rat, but not the mouse, there are some indications that the Ca^2+^ clock could be involved in the training-induced bradycardia: there was a significant downregulation of Ca^2+^-handling genes known to have a central role in the Ca^2+^ clock pacemaker mechanism, a trend of downregulation of RyR2 protein expression and a trend of downregulation of Ca^2+^ clock function (Supplementary Fig. 1). mRNA levels of the L-type Ca^2+^ channel α-subunits, Ca_v_1.2 and Ca_v_1.3, were downregulated in the sinus node of the trained rat by 64% and 82%, respectively (Supplementary Table 1). mRNA levels of the L-type Ca^2+^ channel accessory subunits, Ca_v_α2δ1 and Ca_v_α2δ2, were downregulated by similar amounts, that is 65% and 87%, respectively. Ca_v_α2δ subunits control Ca_v_1 subunit targetting to the membrane^40^. These changes are expected to have an impact on pacemaking in the sinus node if the protein levels of these subunits reflect the mRNA levels. Small-conductance Ca^2+^-activated K^+^ channels, SK1–SK3, are expressed in the heart and contribute to the shape and duration of the action potential^41^,^42^,^43^,^44^. In the sinus node of the trained rats, there was significantly reduced expression of *SK1*, but significantly increased expression of *SK2* (Supplementary Table 1); there was also significantly (false discovery rate (FDR)-corrected Limma test*, P*=0.01) increased expression of *SK3* in the right atrial muscle of the trained rats (Fig. 1h). The functional role of the SK channels in pacemaking is not fully understood. However, in the mouse, knockout of SK2 has been reported to cause sinus bradycardia and overexpression of *SK2*, sinus tachycardia^45^. Finally, although the data reported here show that the resting heart rate adaption to training is not the result of changes in resting autonomic tone, it is possible that the altered autonomic tone during exercise triggers the remodelling.

After lifelong physical endurance training, veteran endurance athletes have a higher incidence of sinus node disease and artificial pacemaker implantation than normal individuals^5^,^6^,^7^, and it is likely that this is a consequence of the marked remodelling of the sinus node shown here. It may also help explain syncope in the young athlete^46^. Other rhythm disturbances beset the athlete (for example, atrial fibrillation, heart block^47^,^48^, bundle branch block^48^,^49^ and even sudden cardiac death^50^,^51^) and it is likely that they are a consequence of an analogous remodelling of other parts of the heart: the atrial muscle, atrioventricular node, Purkinje fibres and ventricles (perhaps in combination with a pre-existing heart condition in the case of sudden cardiac death). Exercise is undoubtedly beneficial for the cardiovascular system, but at the same time intense endurance training can have harmful effects, and our findings highlight the fundamental changes taking place.

## Methods

Care and use of laboratory animals conformed to the UK Animals (Scientific Procedures) Act 1986 and European (86/609/CEE) and US (National Institutes of Health (NIH) publication No 85-23) regulations and guidelines. Ethical approval for all experimental procedures was granted by the University of Manchester and the Norwegian University of Science and Technology. Sample sizes were chosen following power calculations using previous comparable data, in order to provide 80% power at the 95% confidence level. For *in vivo* studies, electrophysiological experiments and immunolabelling, only outliers resulting from methodological (standardization) errors were excluded from further analysis. For qPCR involving microfluidic cards, outliers were automatically filtered using RealTime Statminer (v 4.1, Integromics). For qPCR involving 96-well plates, the median absolute deviation method was used to identify outliers.

### Animal models

Six-month-old female Sprague–Dawley rats (Møllegaards Breeding Center Ltd, Denmark; initial body weight, 300–325 g) were arbitrarily assigned to either sedentary or trained groups. The rats had a 12-h:12-h light:dark lighting regime. Rats were trained by aerobic interval training—an uphill running programme described by Wisloff *et al.*^52^ Rats were run on a customized treadmill (at an angle of 25°) in a metabolic chamber to measure the maximal O_2_ uptake (
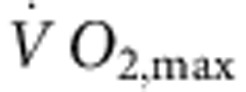
). In brief, the rats were run for 60 min per day (5 days per week) for 12 weeks, alternating between 4 min at 85–90% of 
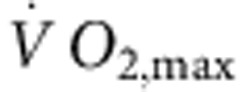
 and 2 min active recovery at 50% of 
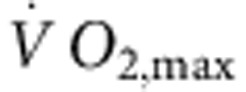
. Ten-week-old male C57/Bl6 mice (Harlan Laboratories; initial body weight, 20–25 g) were arbitrarily assigned to one of three groups: sedentary, trained and detrained. Mice were housed five per cage in a temperature-controlled room (22 °C) with a 12 h:12 h light:dark lighting regime and free access to food and water. Mice in the trained and detrained groups were subject to a swimming programme as described by Liu *et al.*^53^ In brief, mice were swam for 60 min twice a day (7 days per week) for 4 weeks in tanks with a surface area of 32,500 cm^2^, depth of 35 cm and water temperature of 30–32 °C. The mice were initially exercised for 10 min twice a day and the duration of exercise was increased in daily increments of 20 min, finally reaching 60 min. This duration of exercise was maintained until the end of the study. At the end of exercise, mice were carefully towel-dried and kept in a drying enclosure maintained at 30 °C for ~30 min. All mice were able to complete this course of training. After the last training session, the detraining cohort was submitted to detraining for 2 weeks in which physical activity was restricted to the space of the cage. Age- and weight-matched sedentary control mice were handled daily.

### ECG recordings from conscious rats

In rats, ECGs were recorded using a telemetry system (Data Sciences, St. Paul, MN, USA) comprising implantable telemetry devices (ETA-F20 or ETA-F10), receivers (RPC-1), a data exchange matrix and an acquisition and storage system (P3 Plus version 4.8, Data Sciences). The devices were surgically implanted into the peritoneal cavity and electrodes were arranged subcutaneously above and below the heart. Surgery was done under 2–3% isoflurane anaesthesia. Small and light devices designed for mice were used to minimize the impact of implantation and prevent any discomfort for the rats during training. Training started after 1 week of recovery from surgery. The devices were turned on using a magnetic switch and the ECG was recorded in conscious, unrestrained rats. Recordings obtained during the dark cycle of the lighting regime were used for data analysis.

### ECG recordings from conscious mice

ECGs were recorded non-invasively in mice using the ECGenie recording enclosure (Mouse Specifics, Inc., Boston, MA, USA) described by Chu *et al.*^54^ In brief, the ECGenie system comprises a platform with embedded paw-sized AgCl ECG electrodes connected to an amplifier (e-MOUSE). Signals were collected and analysed using PowerLab and LabChart (ADInstruments, version 7). A peak detection algorithm on LabChart enabled R-wave identification using Fourier analysis and linear time-invariant digital filtering of frequencies below 3 Hz and above 100 Hz to minimize environmental signal disturbances. Each mouse was acclimatized to the setup for 10 min before data collection. Only data from continuous recordings were used in the analysis. The signals were digitized at a sampling rate of 2 kHz. To evaluate the effects of ivabradine on heart rate, ECGs were recorded as described above 30 min after administration of 6 mg kg^−1^ body weight of ivabradine hydrochloride (Sigma-Aldrich). Vagal and sympathetic tone were evaluated based on heart rate changes because of sequential pharmacological block according to previously described methods^55^. Mice (*n*=6/6) were given intraperitoneal propranolol (1 mg kg^−1^) and ECGs were recorded for 15 min following which atropine (0.5 mg kg^−1^) was administered to achieve complete autonomic block, and the ECG was recorded for another 15 min. Vagal tone was defined as the difference between the heart rate after propranolol alone and the heart rate after propranolol and atropine. Sympathetic tone was assessed in a separate cohort of mice (*n*=6/6). Atropine (0.5 mg kg^−1^) was administered intraperitoneally. Ten minutes later sympathetic activity was blocked with propranolol (1 mg kg^−1^). Sympathetic tone was calculated as the difference between the heart rate after atropine alone and the heart rate after atropine and propranolol. After drug administration, once a steady state had been reached, the heart rate was measured over 100 consecutive beats and the average was calculated. The route and dose of atropine and propranolol used in this study have been previously verified to produce complete autonomic block^56^,^57^,^58^. The time course of the effects of atropine, propranolol and ivabradine was studied separately in five age-matched C57BL6J mice. Single doses of atropine (0.5 mg kg^−1^), propranolol (1 mg kg^−1^) or ivabradine (6 mg kg^−1^) were injected into conscious mice on separate occasions. Heart rate data were collected using the ECGenie system (as described above) at different time points after injection: baseline, 1, 5, 10, 20, 30, 40, 50 and 60 min (and in the case of ivabradine at 70 and 80 min). The maximal effect of atropine was reached in 5–7 min after injection and sustained for ~15 min. Propranolol required ~10 min to reach maximal effect, with the effect maintained for ~50 min. Ivabradine reached maximal effect at 25–30 min after injection and its maximal effect was maintained for a further 35–40 min. These data were used in designing the experimental protocol for experiments on autonomic tone and to study the effect of ivabradine under complete autonomic block, in which simultaneous maximal effects of drugs were required.

### ECG recordings from anaesthetized mice

After the procedure above, anaesthesia was induced in mice with 5% isoflurane (Isoflo, Portugal) in 100% O_2_ with a delivery rate of 5 l min^−1^ until loss of the righting reflex. After induction, the animals were moved to a homeothermic blanket and placed in dorsal recumbence. Anaesthesia was then maintained with isoflurane in 100% O_2_ with a flow of 1.5 l min^−1^ administered by means of a facemask connected to a coaxial circuit (Fluovac anaesthetic mask, Harvard Apparatus, Reagente 5, Porto, Portugal). The ECG was recorded using electrodes attached to the two front paws and the back right paw (looking at the belly of the mouse). RR intervals were recorded using a PowerLab/4SP with a ML135 Dual Bio amplifier (ADInstruments). After recording the basal heart rate, ivabradine (6 mg kg^−1^) was administered intraperitoneally. Twenty minutes after ivabradine administration, mice were given propranolol and atropine to study the effects of ivabradine under total autonomic block. After the administration of propranolol and atropine, the heart rate was recorded for 20 min. Once again, once a steady state had been reached, the RR interval was measured over 100 consecutive beats and the average was calculated. Following this, the mice were killed.

### Tissue electrophysiology

The beating rate of the isolated sinus node was determined by extracellular potential recording as described by Yamamoto *et al.*^59^ In brief, animals were weighed and then killed by cervical dislocation. The heart was rapidly removed and placed in Tyrode solution containing (in mM) 100 NaCl, 4 KCl, 1.2 MgSO_4_, 1.2 KH_2_PO_4_, 1.8 CaCl_2_, 25 NaHCO_3_ and 10 glucose. The solution was bubbled with 95% O_2_ and 5% CO_2_ to give a pH of 7.4. A right atrial preparation encompassing the sinus node and including the superior vena cava was rapidly dissected and superfused with warm (37 °C) Tyrode solution at a rate of 10 ml min^−1^ for electrophysiological recordings. Extracellular potentials were recorded from the sinus node preparation using bipolar electrodes fabricated from two stainless steel wires, 100 μm, in diameter. These recording electrodes interfaced with a Neurolog system (Digitimer) with low-pass/high-pass filters adjusted to optimize the signal-to-noise ratio. Extracellular potentials were continuously recorded using a PC with a PowerLab and LabChart version 7 software (ADInstruments). The calculated rate was averaged over 500 beats. The effect of 2 mM CsCl (Sigma-Aldrich) on the beating rate was studied. After dissection, the preparation was allowed to stabilize, following which the superfusing solution was changed to Tyrode solution containing CsCl. After 15 min of treatment, the rate was recorded for 5 min. The preparation was then washed for 20 min, during which the beating rate approached baseline values. The calculated rate was averaged over 500 beats.

### Sinus node cell isolation and patch-clamp electrophysiology

Mice were killed by cervical dislocation. After quick removal of the heart, the sinus node tissue was dissected out and strips of nodal tissue were dissociated into single cells by a standard enzymatic and mechanical procedure^24^. The enzymatic solution contained collagenase IV (224 U ml^−1^, Worthington), elastase (1.42 U ml^−1^, Sigma-Aldrich) and protease (0.45 U ml^−1^, Sigma-Aldrich)^18^. Isolated sinus node cells were stored at 4 °C for the day of the experiment. *I*_f_ was recorded using a patch electrode in whole-cell mode during superfusion of a Tyrode solution containing (mM): 140 NaCl, 5.4 KCl, 1.8 CaCl_2_, 1 MgCl_2_, 5 HEPES-NaOH, 10 D-glucose, pH 7.4. BaCl_2_ (1 mM) and MnCl_2_ (2 mM) were added to improve dissection of *I*_f_ from other components. Temperature was 35±0.5 °C. The pipette solution contained (in mM) 130 K-aspartate, 10 NaCl, 2 CaCl_2_ (pCa=7), 2 MgCl_2_, 10 HEPES, 5 EGTA, 2 ATP(Na_2_), 0.1 GTP, 5 creatine phosphate, pH 7.2. To obtain current densities, currents were measured during steps to the range −55/−135 mV from a holding potential of −35 mV and normalized to cell capacitance. Activation curves were obtained by applying two-step protocols with a first step to a test voltage in the range −35/−135 mV and a second step to −135 mV to measure the residual current activation. The duration of test steps varied from 7 s at −35 mV to 1.2 s at −135 mV, so as to allow full current activation at each voltage. Tail currents measured at −135 mV were normalized to maximum amplitude and were plotted to obtain the activation variable and averaged. Mean data points were fitted with the Boltzmann distribution: *y*=(1/(1+exp((*V*−*V*_1/2_)/*s*), where *V* is voltage, *y* fractional activation, *V*_1/2_ the half-activation voltage and *s* the inverse-slope factor. Time constants of activation and deactivation were determined by fitting with a single exponential curve the early time course of the current activated by hyperpolarization, and of the current traces at each test voltage after activation by a prepulse to −125 mV, respectively. The initial delay and any late slow activation were ignored. The fully activated *I*_f_ current–voltage relationship was measured according to a previously developed protocol^60^; pairs of steps to a given test voltage were applied in sequence from two reference voltage levels, one where the current is fully activated (−125 mV) and one where the current is fully deactivated (−35 mV); the difference between tail amplitudes on clamping at the same test potential from the two reference levels (measured between arrows as in Fig. 3) represents the fully activated current amplitude at the test potential itself. Data were acquired at 1 kHz using an Axopatch 200 amplifier and pClamp 8 (Molecular Devices, Sunnyvale, CA, USA). Data were analysed off-line using Clampfit 10 (Molecular Devices), Origin 8 (Origin Lab Corp., Northampton, MA, USA) and GraphPad Prism6.

### Immunohistochemistry

Immunohistochemistry was carried out using established methods as described previously^61^. Briefly, sinus node preparations were dissected as described above, immersed in optimal cutting temperature compound and frozen in liquid N_2_. Frozen sections (20 μm) were fixed in 10% formaldehyde, permeabilized with 0.1% Triton X-100 for 30 min, blocked with 1% bovine serum albumin and incubated with anti-HCN4 raised in rabbit (Millipore, AB5808; 1:100). Finally, sections were incubated with fluorescein isothiocyanate (FITC)- (Millipore, AP182F; 1:100) or CY3- (Millipore, AP182C; 1:400) conjugated secondary antibodies raised in rabbit. Immunofluorescent labelling was carried out in batches with each batch comprising four slides from sedentary animals and four slides from trained animals with 2–3 sections per slide. The sections were from approximately the same level in the sinus node. Antibody concentrations were optimized beforehand. In addition, an extra slide (either control or trained) was included in each batch to standardize laser settings. A slide processed identically, with the exception of primary antibody, was also included in each batch for autofluorescence correction. Images were collected on a Leica TCS SP5 AOBS upright confocal using a × 63/1.40 HCX PL Apo objective and a × 2 field zoom. The confocal settings were as follows: pinhole, 1 airy unit; scan speed, 1 kHz unidirectional; format 512 × 512. Images were collected using the following settings: FITC—488 nm excitation and 494–530 nm emission, and Cy3—594 nm excitation and 640–690 nm emission. When acquiring three-dimensional optical stacks, the confocal software was used to determine the optimal number of Z sections. As planes were acquired with 0.8 μm spacing, measuring 25 planes gave a *z* distance of 20 μm. Maximum intensity projections of three-dimensional stacks are shown in the Results section. Five high power images were obtained from each animal for each antibody. HCN4 or RyR2 immunofluorescence was quantified by measuring the integrated densities in the sinus node region of sections processed at the same time. A 400 × 400 pixel rectangle was drawn in the region of interest and the integrated density of fluorescence was measured within this area in all stacks. To normalize each sample for background, an automatic threshold level was set using the Stack Threshold plugin on ImageJ (NIH, Bethesda, MD, USA) that analyses the image distribution of pixel intensities (image histogram) and sets a threshold value that separates a background cluster from the foreground pixels.

### RNA isolation and qPCR

Punch biopsies were taken from the sinus node preparation described above using a 1-mm diameter punch (Miltex, York, PA, USA). Biopsies were collected from the sinus node approximately at the level of the main branch from the crista terminalis and the neighbouring right atrial free wall. Biopsies were frozen in liquid N_2_ and stored at −80 °C until use. Total RNA was isolated from the biospies using the RNeasy Micro kit (Qiagen) according to the manufacturer’s instructions. RNA purity and quantity was determined using a NanoDrop ND-1000 spectrophotometer (NanoDrop Technologies, Wilmington, DE, USA). Samples with an OD_260/280_ reading between 1.8 and 2.1 were used. First strand cDNA was synthesized using Superscript II reverse transcriptase (Invitrogen, Carlsbad, CA, USA). qPCR was performed using an ABI Prism 7900 HT Sequence Detection System (Applied Biosystems/Life Technologies Corporation, Carlsbad, CA, USA).

### qPCR for the rat

For the rat, PCRs were carried out using customized TaqMan Array for Gene Expression microfluidic cards (Applied Biosystems, cat. no. 4342259; format 96A). Each microfluidic card consisted of 384 wells preloaded with fluorogenic TaqMan probes and primers organized in eight ports of 48 genes each. Ninety-six transcripts were measured in each sample. The transcripts studied are listed in Supplementary Tables 1 and 2. cDNA (150 ng) was added to each port with 1 × TaqMan Universal Master Mix (Applied Biosystems). Thermal cycling conditions were 50 °C for 2 min and 94.5 °C for 10 min, followed by 40 cycles at 97 °C for 30 s and 59.7 °C for 1 min. Data were collected with ABI Prism 7900 HT Sequence Detection System software (SDS 2.3). Data were analysed using RealTime Statminer, which enabled advanced filtering of outlier genes and samples and selection of 18S as the optimal endogenous control using algorithms such as geNorm, NormFinder and Minimum Variance Median. Transcript expression levels were calculated using the Δ*C*_t_ method that corrects for variations in input RNA by normalization of the abundance of the transcript of interest to the abundance of a reference transcript. Samples presenting with *C*_t_ (threshold cycle) values ≥38 were excluded from further analysis for lack of reproducibility. Data in graphs represent the Δ*C*_t_ values transformed to log_2_. Statistical significance was tested using the non-parametric Limma test as well as the Benjamini–Hochberg FDR, both of which have been previously validated for multiple test correction in microarray data analysis^62^,^63^. The FDR estimates the number of false positives from the total number of reported positives and in this study a FDR-corrected *P* value <0.05 was used to denote significance. This indicates a FDR of 5%. In Supplementary Table S1, the FDR-adjusted *P* value is shown.

### qPCR for the mouse

For the mouse, PCRs were carried out using 96-well plates. The reaction mixture comprised 1 μl of cDNA, 1 × Qiagen assay (HCN4, QT00268660; Tbx3, QT00147042; RyR2, QT00153223; SERCA2a, QT00149121; phospholamban, QT00254366; CASQ2, QT01058078; and NCX1, QT01044862), 1 × SYBR Green Master Mix (Applied Biosystems) and DNase-free water. All samples were run in duplicate. The reaction conditions were: denaturation step of 95 °C for 10 min followed by 40 cycles of amplification and quantification steps of 95 °C for 30 s, 60 °C for 30 s and 72 °C for 1 min. The melt curve conditions were: 95 °C for 15 s, 60 °C for 15 s and 95 °C for 15 s. mRNA expression normalized to the expression of the housekeeper, 18S, was calculated using the Δ*C*_t_ method.

### miRNA analysis

Sinus node expression of miR-1 was measured using miRCURY LNA (Locked Nucleic Acid) Universal RT microRNA PCR setup (Exiqon, Denmark) using the manufacturer’s instructions for cDNA synthesis and qPCR. In brief, RNA was extracted from sinus node biopsies (Qiagen Micro kit) following which mature miRNAs were tailed with a poly(A) sequence at the 3′ end. Reverse transcription into cDNA entailed application of a universal PolyT primer with a 3′ end degenerate anchor and a 5' end universal tag. The cDNA products were subsequently diluted 80-fold and quantified using SYBR green based qPCR and a LNA-enhanced miRNA-specific primer for miR-1 (mouse: hsa-miR-1, target sequence: 5′-UGGAAUGUAAAGAAGUAUGUAU-3′, 204344; rat, rno-miR-1, target sequence: 5′-UGGAAUGUAAAGAAGUGUGUAU-3′, 205104, Exiqon). The qPCR was run on the ABI Prism 7900 HT Sequence Detection System using the following thermal cycling parameters: 95 °C for 10 min, 45 amplification cycles at 95 °C for 10 s and 60 °C for 1 min followed by 95 °C for 15 s, 60 °C for 15 s and 95 °C for 15 s to generate a melt curve. Samples were run in duplicate. Raw *C*_t_ values were calculated as recommended by Exiqon using RQ Manager (v1.2.1, Applied Biosystems) with manual settings for threshold and baseline. Expression of miRNA was calculated by the Δ*C*_t_ method and normalization to expression of RNU1A1, which was determined as the optimal endogenous control using the algorithm geNorm (qBase^plus^, version 2.0, Biogazelle, Belgium).

## Additional information

**How to cite this article**: D’Souza, A. *et al.* Exercise training reduces resting heart rate via downregulation of the funny channel HCN4. *Nat. Commun.* 5:3775 doi: 10.1038/ncomms4775 (2014).

## Supplementary Material

Supplementary Information Supplementary Figures 1-4 and Supplementary Tables 1-4
