# Supplementary material for: Exercise training reduces resting heart rate via downregulation of the funny channel HCN4
Source: Nat Commun. 2014 May 13;5:3775. doi: 10.1038/ncomms4775 (PMC4024745; doi:10.1038/ncomms4775)
Supplement: Supplementary Information — Supplementary Figures 1-4 and Supplementary Tables 1-4 [file ncomms4775-s1.pdf]

SUPPLEMENTARY INFORMATION

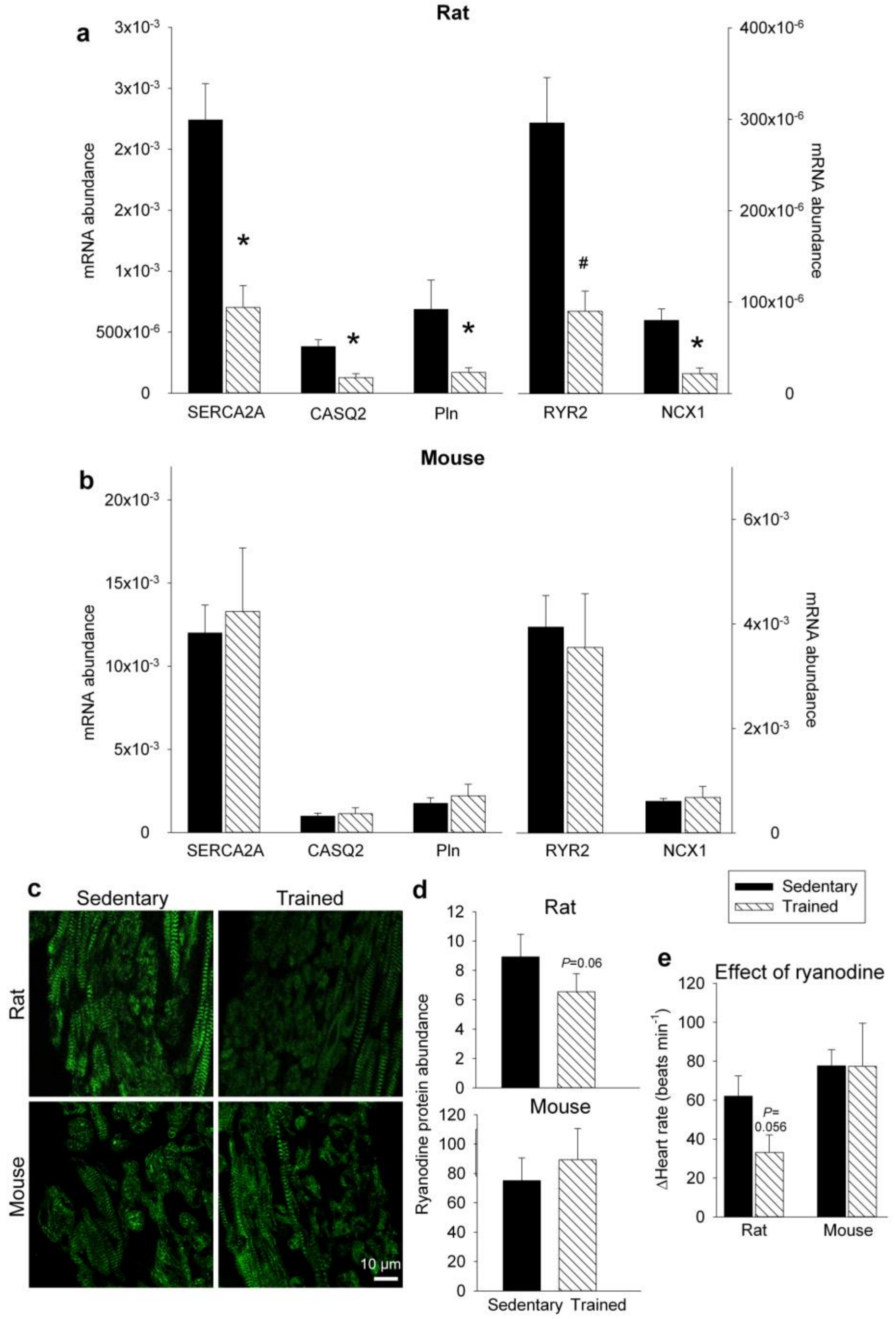

Supplementary Figure 1. Is the Ca^{2+} clock altered by training? a and b, Mean (+SEM) mRNA expression of key proteins comprising the Ca^{2+} clock in the rat (**a**; n=5/8) and mouse (**b**; n=6/7). mRNA expression was normalised to 18S. **c**, Representative images of RyR2 immunolabelling (green signal) in the sinus node of sedentary and trained rats (top) and mice (bottom). Scale bar, 10 µm. **d,** Mean (+SEM) RyR2 protein expression in rats (n=4/4) and mice (n=4/4). **e**, Mean (+SEM) change in the spontaneous rate of the isolated sinus node on disruption of the Ca^{2+} clock by 2 µM ryanodine in rats (n=7/7) and mice (n=6/6). For **a** and **b**, statistical significance of differences between sedentary and trained animals was tested using the non-parametric limma test and Benjamini-Hochberg False Discovery Rate (FDR) **(***$P<0.05$; #$P<0.2$**).** For **d** and **e**, statistical significance was tested using Student's *t* test (*$P<0.05$). Normal distribution of data was tested using the Shapiro-Wilk W test and equal variance was tested using the *F* test.

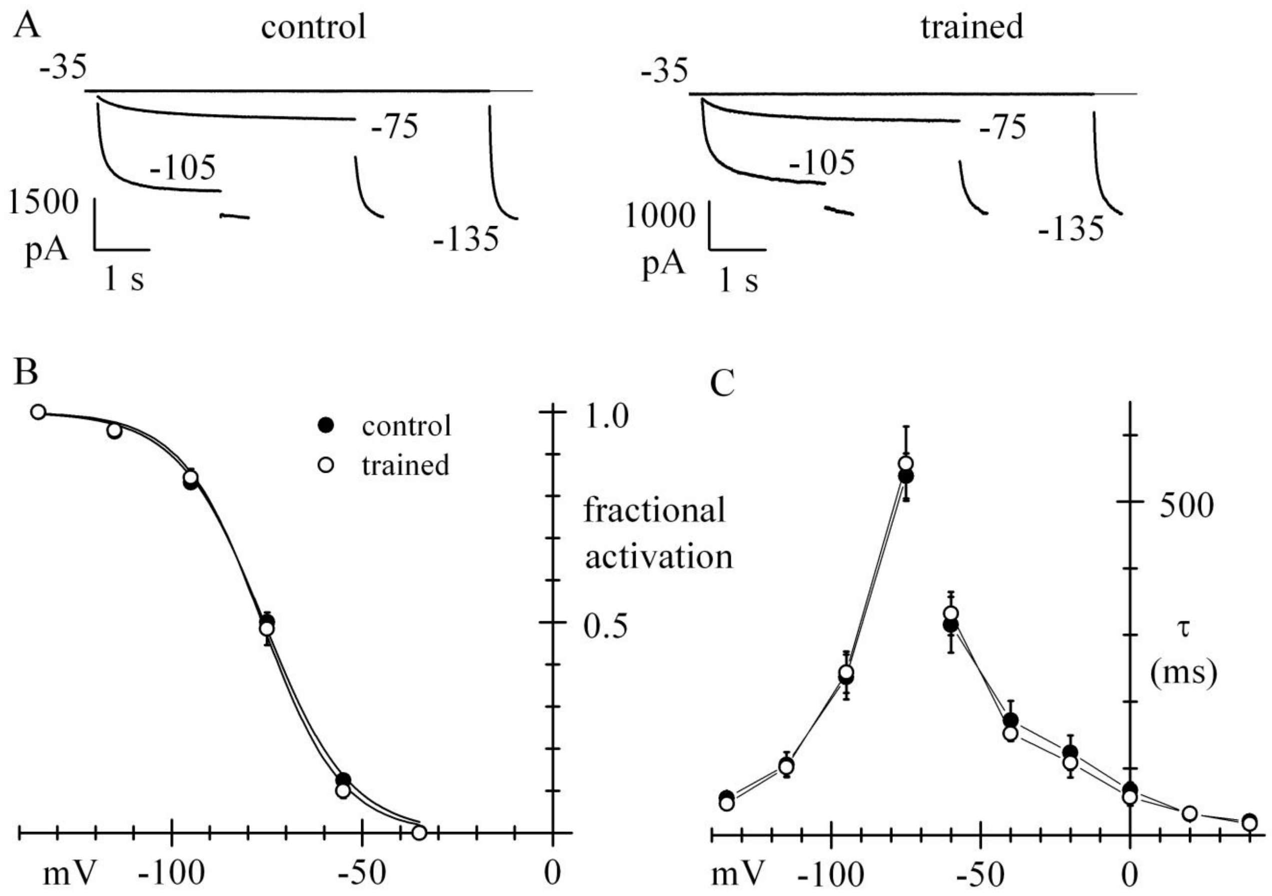

Supplementary Figure 2. I_f kinetics are not modified by training. a, Sample current traces recorded with a two-step protocol to measure the activation curve from single sinus node cells isolated from sedentary (left) and trained (right) mice. Only records at test voltages of -35, -75 and -105 mV are shown. **b**, Mean (±SEM) steady-state activation curves for sedentary (n=9/5 cells/mice, filled circles) and trained (n=10/5, empty circles) mice. Data were fitted with the Boltzmann equation (solid lines); half-activation voltages and inverse-slope factors were: -75.8 and 11.2 mV for sedentary mice and -76.3 and 10.5 mV for trained mice. For both groups the individual activation curves were each fit to the Boltzmann equation, and the calculated midpoint (V1/2) and slope factor (s) compared using the Student t-test. Mean values obtained were not significantly different (*P*>0.05). **c**, Mean (±SEM) activation and deactivation time constant curves for sedentary and trained mice (symbols as in **b**). Data negative to -70 mV are activation time constants (n=8/5 for sedentary and n=10/5 for trained mice) and data positive to -70 mV are deactivation time constants (n=9/5 for sedentary and n=8/5 for trained mice). Curves through points. Activation/deactivation kinetics of training mice are not statistically different than those of control mice (Student t-test, *P*>0.05).

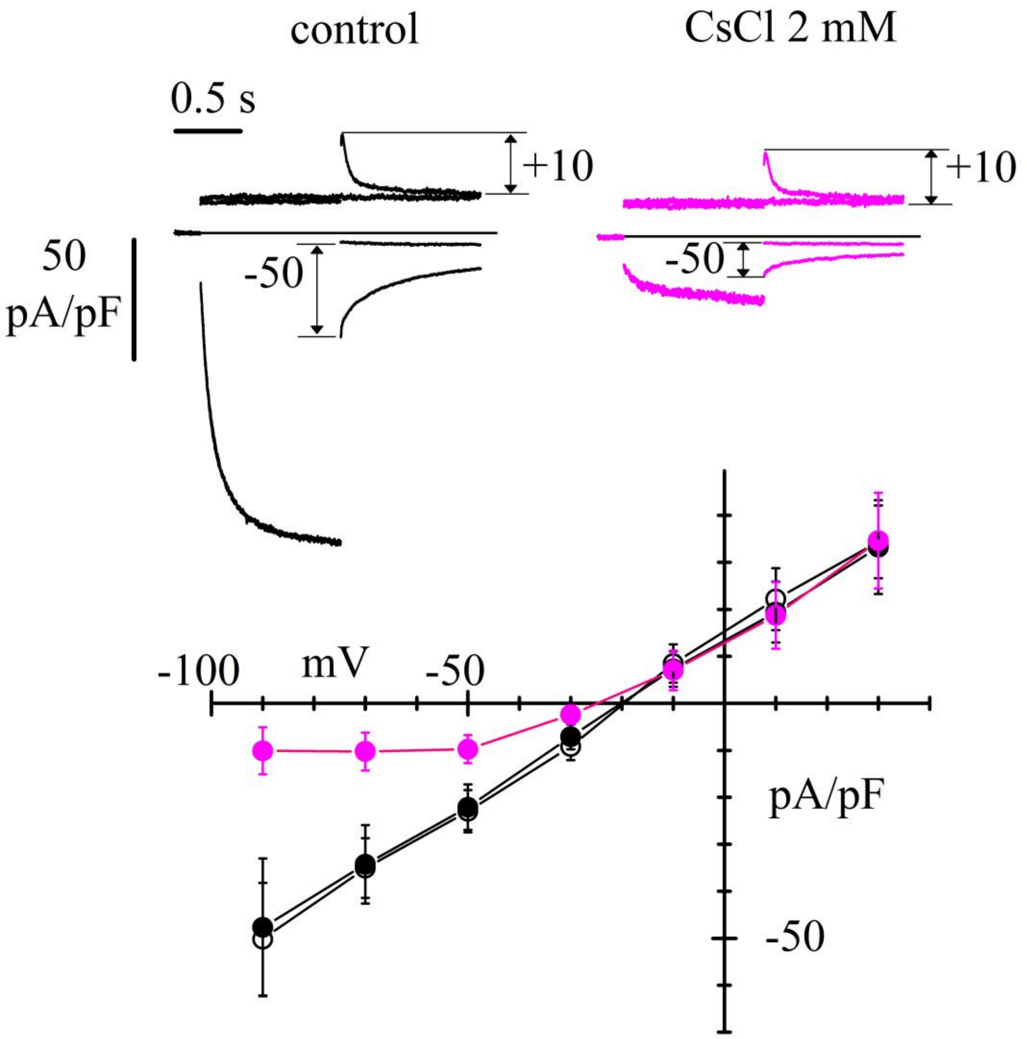

Supplementary Figure 3. Action of 2 mM CsCl on I_f recorded from SAN cells isolated from sedentary mice. Top, sample current traces recorded with the protocol used to measure the fully activated IV relationship in control solution (left) and in the presence of 2 mM CsCl (right). Conditioning activating/deactivating voltages were -125 and +10 mV, and pairs of steps to the same test voltage (range, -90 through +30 mV) were applied in sequence from these two levels. Records are shown for the test voltages of -50 and +10 mV. Bottom, mean (±SEM) fully-activated IV relationship in control (black circles, n=6), after addition of 2 mM CsCl (magenta circles, n=6) and after return to control (empty circles, n=5) is shown. The voltage dependence of block is apparent from the outward-rectifying shape of the I-V curve in the presence of Cs^+. In the negative voltage region of the I-V curve, the block is characterised by an increased current block at more negative voltages. The blockade caused by 2 mM Cs^+ was 78.8%, 70.0% and 55.8% at -90, -70 and -50 mV, respectively. Current amplitudes in the range -90 mV to -30 mV were significantly reduced by 2 mM CsCl ($P<0.05$, Paired Student's t-test).

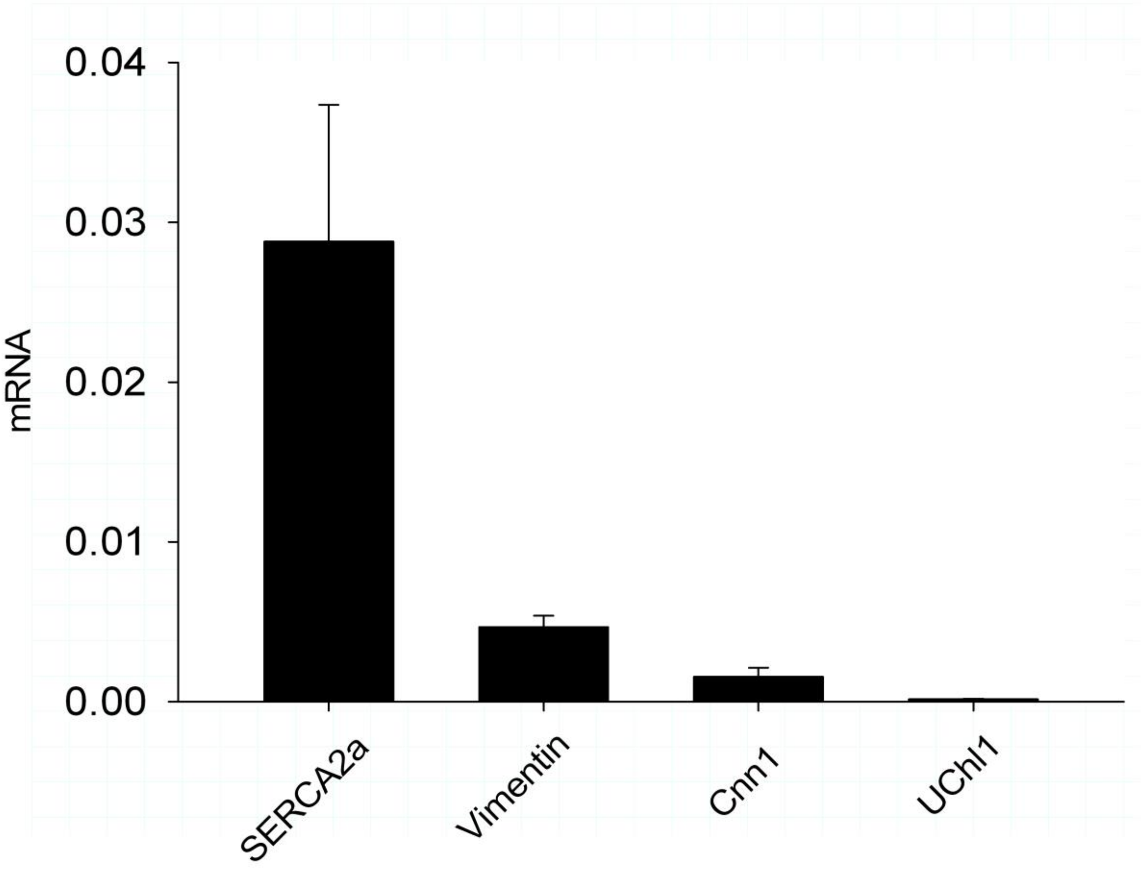

Supplementary Figure 4. mRNA expression of cell type markers in mouse sinus node biopsies. mRNA expression was normalised to expression of 18S mRNA. The expression of the cardiomyocyte marker, SERCA2a, was 6.2-fold higher than the expression of a fibroblast marker, vimentin; 18.5-fold higher than the expression of a vascular smooth muscle cell marker, Cnn1; and 189-fold higher than the expression of a neuronal marker, Uchl1. Means+SEM (n=5) shown.

Supplementary Table 1 (part 1). mRNAs in the rat sinus node that changed significantly in response to training.

mRNA	Sedentary (mean±SEM)	Trained (mean±SEM)	FDR adjusted P value	Trained/sedentary (%)	n	Applied Biosystems Assay ID
**HCN channels**						
HCN1	0.9 ± 0.2	0.3 ± 0.1	0.010	30%	5/8	Rn00670384_m1
HCN4	19.4 ± 3.5	6.3 ± 1.2	0.010	32%	5/8	Rn00572232_m1
**Na^+ channels**						
$Na_v1.5$	124 ± 15.4	37.5 ± 9.4	0.013	30%	5/8	Rn00565502_m1
$Na_v2.1$	20.9 ± 3.1	7.7 ± 2.2	0.010	37%	5/8	Rn00581647_m1
**Ca^{2+} channels**						
$Ca_v1.2$	14.4 ± 2.4	5.3 ± 1.3	0.016	36%	5/8	Rn00709287_m1
$Ca_v1.3$	0.022 ± 0.012	0.004 ± 0.001	0.039	18%	5/8	Rn00568820_m1
$Ca_v3.1$	34.0 ± 12.6	4.7 ± 1.1	0.007	14%	5/8	Rn00581051_m1
$Ca_v3.2$	12.0 ± 4.9	1.2 ± 0.3	0.007	10%	5/8	Rn01460348_m1
$Ca_v\alpha2\delta1$	8.3 ± 2.2	2.9 ± 0.6	0.024	35%	5/8	Rn01442580_m1
$Ca_v\alpha2\delta2$	19.5 ± 7.1	2.6 ± 0.6	0.007	13%	5/8	Rn00457825_m1
$Ca_v\beta2$	9.4 ± 2.2	2.1 ± 0.5	0.007	22%	5/8	Rn00587789_m1
**Transient outward K^+ channels**						
$K_v1.4$	2.8 ± 0.8	1.4 ± 0.6	0.020	48%	5/7	Rn02532059_s1
KChIP2	42.0 ± 10.2	12.3 ± 2.8	0.019	29%	5/8	Rn01411451_m1
**Delayed rectifier K^+ channels**						
$K_v1.2$	7.3 ± 1.7	2.0 ± 0.8	0.013	28%	5/7	Rn02094595_s1
ERG1	6.9 ± 2.1	2.0 ± 0.4	0.020	28%	5/8	Rn00588515_m1
**Inward rectifier K^+ channels**						
$K_{ir}2.1$	16.4 ± 4.7	3.3 ± 0.7	0.010	20%	5/8	Rn00568808_s1
$K_{ir}2.2$	17.6 ± 5.4	3.9 ± 1.0	0.010	22%	5/8	Rn02533449_s1
$K_{ir}3.1$	89.4 ± 24.6	27.8 ± 7.1	0.023	31%	5/8	Rn00434617_m1
$K_{ir}3.4$	47.3 ± 10.7	16.6 ± 3.4	0.022	35%	5/8	Rn01789221_mH
$K_{ir}6.1$	6.9 ± 0.9	2.2 ± 0.6	0.010	32%	5/8	Rn01492857_m1
$K_{ir}6.2$	29.0 ± 5.8	8.6 ± 1.8	0.010	29%	5/8	Rn01764077_s1
SUR1	16.5 ± 3.7	4.2 ± 0.9	0.019	25%	5/8	Rn00564778_m1
SUR2	40.0 ± 7.8	8.2 ± 1.7	0.007	20%	5/8	Rn00564842_m1
**Miscellaneous K^+ channels**						
SK1	1.3 ± 0.7	0.5 ± 0.1	0.028	40%	5/8	Rn00570904_m1
SK2	0.3 ± 0.1	0.9 ± 0.5	0.010	258%	5/8	Rn00570910_m1
TASK1	79.9 ± 13.9	81.6 ± 12.8	0.027	102%	5/8	Rn00583727_m1
TRPC1	5.4 ± 1.4	1.2 ± 0.3	0.007	21%	5/8	Rn00585625_m1
**Cl^- channels**						
Chloride channel 2	1.7 ± 0.5	1.8 ± 0.3	0.013	107%	5/8	Rn00567553_m1
Chloride channel 3	7.3 ± 1.6	16.0 ± 8.5	0.007	218%	5/8	Rn01535195_m1
**Gap junction channel**						
Cx45	8.7 ± 2.0	2.8 ± 0.8	0.011	32%	5/8	Rn01750705_m1
**Na^+-K^+ Pump**						
Na^+-K^+ pump α1 subunit	351.0 ± 87.2	97.9 ± 25.1	0.010	28%	5/8	Rn01533986_m1
Na^+-K^+ pump α2 subunit	35.2 ± 3.5	15.4 ± 4.5	0.031	44%	5/8	Rn00560789_m1
Na^+-K^+ pump β1 subunit	215.0 ± 41.8	63.4 ± 14.3	0.011	29%	5/8	Rn00565405_m1
**Intracellular Ca^{2+}-handling molecules**						
NCX1	80.0 ± 12.7	21.9 ± 6.2	0.007	27%	5/8	Rn00570527_m1
SERCA2a	2240.0 ± 299.0	703.0 ± 180.0	0.012	31%	5/8	Rn00568762_m1
Phospholamban	686.0 ± 240.0	169.0 ± 38.5	0.010	25%	5/8	Rn01434045_m1
Calsequestrin 2	381.0 ± 56.6	127.0 ± 32.6	0.010	33%	5/8	Rn00567508_m1
RYR2	296.0 ± 49.6	90.1 ± 22.1	0.013	30%	4/8	Rn01470303_m1
RYR3	2.6 ± 0.4	1.0 ± 0.3	0.010	37%	5/8	Rn01328415_g1
IP_3 receptor 1	14.3 ± 2.9	5.0 ± 1.4	0.011	35%	5/8	Rn01425738_m1
IP_3 receptor 2	5.1 ± 0.9	2.0 ± 0.6	0.012	40%	5/8	Rn00579067_m1
IP_3 receptor 3	5.6 ± 1.2	1.5 ± 0.5	0.011	27%	5/8	Rn00565664_m1
**Receptors**						
α1A/1C adrenergic receptor	7.4 ± 3.0	1.4 ± 0.3	0.012	19%	5/8	Rn00567876_m1
α1B adrenergic receptor	8.5 ± 1.5	2.5 ± 0.4	0.013	29%	5/8	Rn01471343_m1
β1 adrenergic receptor	44.3 ± 8.1	12.8 ± 2.6	0.013	29%	5/8	Rn00824536_s1
β2 adrenergic receptor	5.8 ± 1.6	1.8 ± 0.5	0.017	30%	5/7	Rn00560650_s1
M2 muscarinic receptor	41.6 ± 10.2	15.3 ± 5.1	0.013	37%	5/8	Rn02532311_s1
**Extracellular matrix components**						
Collagen type 1 a	25.4 ± 5.2	9.0 ± 2.3	0.020	36%	5/8	Rn01463848_m1
Collagen type 3 a	226.0 ± 29.4	68.5 ± 19.4	0.011	30%	5/7	Rn01437681_m1
TGFβ3	5.5 ± 1.1	1.8 ± 0.4	0.010	32%	5/8	Rn01475964_m1

mRNA expression ($\times10^6$) shown relative to that of the housekeeper gene, 18S.

Supplementary Table 1 (part 2). mRNAs in the rat sinus node that changed significantly in response to training.

mRNA	Sedentary (mean±SEM)	Trained (mean±SEM)	FDR adjusted P value	Trained/sedentary (%)	n	Applied Biosystems Assay ID
**cAMP pathway**						
Adenylate cyclase 4	6.4 ± 1.3	2.0 ± 0.5	0.010	31%	5/8	Rn00570644_m1
Adenylate cyclase 4	52.4 ± 12.2	13.0 ± 3.0	0.007	25%	5/8	Rn00575059_m1
Protein kinase A catalytic subunit α	99.5 ± 28.0	30.8 ± 7.8	0.010	31%	5/8	Rn01432302_m1
Protein kinase A catalytic subunit β	17.5 ± 3.9	4.7 ± 1.2	0.013	27%	5/8	Rn01293014_m1
cAMP-dependent protein kinase type I a regulatory chain	106.0 ± 25.9	31.2 ± 6.0	0.010	29%	5/8	Rn00566036_m1
cAMP-dependent protein kinase type 2 a regulatory chain	34.3 ± 9.6	8.2 ± 2.0	0.011	24%	5/8	Rn00709403_m1
Ca^{2+}/calmodulin-dependent protein kinase II	16.8 ± 2.9	4.0 ± 0.9	0.007	24%	5/8	Rn00560913_m1
Phosphodiesterase 2a	9.9 ± 0.9	3.5 ± 0.8	0.010	36%	5/8	Rn01648917_m1
Phosphodiesterase 4d	13.5 ± 3.1	2.8 ± 0.6	0.007	21%	5/8	Rn00566798_m1
**Miscellaneous**						
Cardiac myosin heavy chain α6	5420 ± 2500	1900 ± 1200	0.019	35%	5/8	Rn00691721_g1
Atrial natriuretic peptide precursor	24300 ± 23400	2110 ± 1550	0.019	9%	5/7	Rn00561661_m1
Tbx3	8.9 ± 4.1	1.0 ± 0.3	0.010	11%	5/8	Rn00710902_m1

mRNA expression ($\times 10^6$) shown relative to that of the housekeeper gene, 18S.

Supplementary Table 2. mRNAs in the rat sinus node that were not significantly altered by training.

mRNA	Sedentary (mean±SEM)	Trained (mean±SEM)	FDR Adjusted *P* value	n	Applied Biosystems Assay ID
**HCN channels**					
HCN2	2.38±0.60	1.12±0.30	0.091	5/8	Rn01408575_gH
**Na^+ channels**					
$Na_v1.1$	1.26±0.27	0.616±0.18	0.091	5/8	Rn00578439_m1
$Na_v\beta.1$	56.3±6.65	33.3±10.4	0.105	5/8	Rn00581647_m1
**Transient outward K^+ channels**					
$K_v1.5$	6.51±2.34	1.66±0.40	0.105	5/8	Rn00564245_s1
$K_v4.2$	0.881±0.19	0.619±0.24	0.361	5/8	Rn00581941_m1
$K_v4.3$	0.596±0.19	0.0842±0.02	0.051	5/8	Rn00709609_m1
**Delayed rectifier K^+ channels**					
$K_v2.1$	13.2±2.01	8.17±2.16	0.105	5/8	Rn00755102_m1
**Inward rectifier K^+ channels**					
$K_{ir}2.4$	0.977±0.26	0.447±0.18	0.161	5/8	Rn00821873_m1
K_VLQT1	15±2.98	4.29±1.32	0.102	5/8	Rn00583376_m1
**Miscellaneous K^+ channels**					
SK3	0.3±0.06	1.02±0.32	0.721	5/8	Rn00570912_m1
TWIK 1	1.87±1.72	0.266±0.07	0.892	5/8	Rn00572452_m1
TRPC3	1.04±0.23	0.417±0.14	0.082	5/8	Rn00572928_m1
TRPC6	0.0983±0.05	0.18±0.09	0.166	5/8	Rn00677559_m1
**Gap junction channel**					
Cx43	309±169	4040±3990	0.531	5/8	Rn01433957_m1
Cx40	1.84±0.31	0.631±0.18	0.105	5/8	Rn00570632_m1
Cx30.2	8.67±2.03	2.8±0.75	0.129	5/8	Rn01771737_s1
**Intracellular Ca^{2+}-handling molecules**					
PMCA1	0.303±0.21	2.36±1.59	0.127	4/8	Rn00584038_m1
**Receptors**					
Adenosine α1 receptor	7.38±2.96	1.4±0.34	0.139581	5/8	Rn00567668_m1
α1D adrenergic receptor	0.226±0.098	1.43±0.80	0.051	5/8	Rn00577931_m1
**cAMP pathway**					
cAMP-dependent protein kinase type 2β regulatory subunit	2.15±0.50	5.34±3.09	0.355	5/8	Rn01748544_m1
Phosphodiesterase 1b	0.483±0.12	0.392±0.12	0.301	5/8	Rn00575591_m1
Phosphodiesterase 3b	1.66±0.42	2.5±1.1	0.516	5/8	Rn00568191_m1
**Miscellaneous**					
Brain natriuretic peptide precursor	31.4±59	8.84±8.88	0.504	5/8	Rn00676450_g1

mRNA expression ($\times 10^6$) shown relative to that of the housekeeper gene, 18S.

Supplementary Table 3. Relationship between mRNA expression of key pacemaker components and $\dot{V}O_{2,max}$ in rats (sedentary and trained).

Dependent variable (mRNA)	R^2	*P*
**HCN4**	**0.69**	**0.0008**
**RyR2**	**0.54**	**0.006**
**NCX1**	**0.40**	**0.06**
**SERCA2a**	**0.37**	**0.03**
**$Ca_v1.2$**	**0.36**	**0.03**
**Phospholamban**	**0.34**	**0.04**
$Ca_v3.1$	0.23	0.11
$Ca_v1.3$	0.23	0.18
PMCA1	0.01	0.69

Coefficient of determination (R^2) and *P* values were computed by fitting data with straight lines by linear regression. n=4-5/8 rats. Significant correlations are highlighted in bold. Data are ranked according to the R^2 value.

Supplementary Table 4. Relationship between mRNA expression of key pacemaker components and transcriptional regulators of HCN4 in rats (sedentary and trained).

Independent variable: Tbx3 mRNA

Dependent variable (mRNA)	R^2	*P*
**HCN4**	**0.7**	**0.007**
**RyR2**	**0.54**	**0.006**
**NCX1**	**0.47**	**0.03**
**Phospholamban**	**0.34**	**0.04**
SERCA2a	0.27	0.07
$Ca_v1.3$	0.13	0.32
PMCA1	0.11	0.27
$Ca_v3.1$	0.11	0.28
$Ca_v1.2$	0.1	0.3

Independent variable: NRSF mRNA

Dependent variable (mRNA)	R^2	*P*
**HCN4**	**0.37**	**0.04**
NCX1	0.09	0.42
Phospholamban	0.08	0.42
$Ca_v3.1$	0.08	0.44
RyR2	0.07	0.44
PMCA1	0.06	0.48
$Ca_v1.3$	0.04	0.65
$Ca_v1.2$	0.02	0.65
SERCA2a	0.01	0.73

Independent variable: miR-1

Dependent variable (mRNA)	R^2	*P*
**HCN4**	**0.36**	**0.04**
RyR2	0.19	0.17
NCX1	0.13	0.36
Phospholamban	0.16	0.21
SERCA2a	0.93	0.001
$Ca_v1.2$	0.03	0.61
PMCA1	0.01	0.37
$Ca_v3.1$	0.06	0.5
$Ca_V1.3$	0.0004	0.95

Coefficient of determination (R^2) and *P* values were computed by fitting data with straight lines by linear regression. n=4-5/8 rats. Significant correlations are highlighted in bold. Data are ranked according to the R^2 value.
